# Investigation Into the Predictive Potential of Three-Dimensional Ultrasonographic Placental Volume and Vascular Indices in Gestational Diabetes Mellitus

**DOI:** 10.3389/fendo.2021.689888

**Published:** 2021-06-10

**Authors:** Zhenyan Han, Yuan Zhang, Xuelan Li, Wei-Hsiu Chiu, Yuzhu Yin, Hongying Hou

**Affiliations:** ^1^ Department of Obstetrics and Gynecology, Third Affiliated Hospital of Sun Yat-sen University, Guangzhou, China; ^2^ Health Care Center of Minzhi Community, Shenzhen, China; ^3^ Department of Obstetrics and Gynecology, Guangzhou iBorn Women’s Hospital, Guangzhou, China; ^4^ Department of Ultrasound in Obstetrics and Gynecology, Xuzhou Women and Children’s Peace Hospital, Xuzhou, China

**Keywords:** gestational diabetes mellitus, three-dimensional power Doppler, placental volume, vascularization index, flow index, vascularization flow index

## Abstract

**Background:**

The use of ultrasonography in pregnancies complicated with gestational diabetes mellitus (GDM) can vary according to clinical practice. This study aims to compare the changes of placental volume (PV) and vascular indices measured by three-dimensional (3D) Power Doppler between pregnant women with and without GDM.

**Materials and Methods:**

This was a prospective study of singleton pregnancies who took the early nuchal translucency examination from January 2018 to September 2019. Data on PV and vascular indices including vascularization index (VI), flow index (FI), and vascularization flow index (VFI) between pregnant women with and without GDM were measured by 3D Power Doppler ultrasound machine. Univariate and multivariate logistic regression determined the association between risk factors and GDM. Receiver operating characteristic (ROC) and area under the ROC curve (AUC) were applied to evaluate the diagnostic value of different parameters for GDM.

**Results:**

Of the 141 pregnant women enrolled, 35 developed GDM and 106 did not. The maternal age and gravida in the GDM group were significantly higher than that in the non-GDM group. The PV, VI, FI, and VFI in the GDM group were significantly lower than that in the non-GDM group. There were no significant differences in other clinical parameters between the two groups. After adjustments in multivariate logistic regression analysis, significant differences were observed in VI [odds ratio (OR) = 0.98, 95% confidence interval (CI) = 0.951–1.002], FI (OR = 0.93, 955 CI: 0.86–1.00), and VFI (OR = 0.67, 95% CI = 0.52–0.87). ROC analysis indicated that the combination of maternal age, gravida, PV, and VFI was more accurate as a marker for detecting GDM than the PV, VI, FI, or VFI alone.

**Conclusions:**

The 3D ultrasonography results suggest that PV and vascular indices (VI, FI, and VFI) during the first trimester may serve as potential markers for GDM diagnosis. The combination of maternal age, gravida, and sonographic markers may have good diagnostic values for GDM, which should be confirmed by further investigations.

## Introduction

Gestational diabetes mellitus (GDM) is featured by abnormal glucose intolerance during pregnancy (1). GDM affects about 7% of pregnancies, where advanced maternal age, obesity, previous history of GDM, previously giving birth to macrosomic infants, and family history of diabetes mellitus are regarded as major risk factors (2–4). Oral glucose tolerance test is often used for screening GDM during 24–28 gestational weeks (5, 6). However, GDM screening at 24–28 gestational weeks is too late for the physicians to make dietary or pharmacology therapy, which may ultimately affect placental integrity and fetal growth (7, 8). Thus, identifying novel markers for early screening of GDM is of great clinical significance.

Although clinical manifestations of GDM usually occur in the second or third trimester, evidence of abnormal placental development from as early as the first trimester has been reported, and dysregulation of various hormones and cytokines caused by GDM during early pregnancy has been reported to lead to placental dysfunction (9). Furthermore, increases in inflammatory markers due to persistent hyperglycemia can cause damage within the placenta such as villous maturation, vascularization, and branching (10). Ultrasonography is a non-invasive, readily available tool to examine and assess the fetus, which is helpful in instituting early therapeutic interventions for pregnancies complicated by diabetes (11). Improvements in three-dimensional (3D) ultrasonography have pointed out the role of placental volume (PV) and the potentially associated factors contributing to pregnancy complications. Pala et al., used Virtual Organ Computer-aided AnaLysis (VOCAL) to evaluate PV and placental mean gray value, and found that PV was significantly increased in GDM, whereas mean gray values did not alter (12). Saha et al., observed that the placentae in GDM were significantly bigger in size, weight, volume, area, thickness, diameter, and circumference than those in normal pregnant women, and there was significant increase in villous edema, fibrin deposition, calcification, and congestion of blood vessels in GDM (13). Studies from Wong et al., showed that placental vascular indices can provide an insight into placental vascularization in GDM during early pregnancy, and vascularization flow index (VFI) rather than placental volume may be a sensitive sonographic marker in the first trimester of GDM placentas (14), which still need to be confirmed with larger sample size. Desoye et al., proposed that the placentae in diabetic pregnancies increased levels of thromboxane and tumor necrosis factor alfa leading to vasoconstriction that may contribute to the decrease in vascularization index (VI) and VFI (15).

Based on the above evidence, the association between morphometric changes of placenta and GDM during early pregnancy remains elusive. Therefore, this study aims to determine the PV and vascular indices [vascularization index (VI), flow index (FI), and VFI] by using VOCAL during pregnancy, and to explore the association between these sonographic markers in combination with other clinical parameters and GDM. The present study may identify sensitive sonographic markers for the early diagnosis of GDM, which may be important to avoid the potential complications of GDM.

## Materials and Methods

This prospective study of singleton pregnancies who took the early nuchal translucency examination was performed at Guangzhou iBorn Women’s Hospital from January 2018 to September 2019. A total of 161 pregnant women received ultrasonographic examination during 11^+0^ and 13^+6^ weeks. The inclusion criteria were: (1) gestational age between 11 + ^0^ and 13 + ^6^ weeks; (2) crown-lump length (CRL) between 45 and 80 mm; (3) singleton pregnancy. The exclusion criteria were: (1) fetal chromosomal or structural anomalies detected during karyotyping or sonographic examinations; (2) multifetal pregnancy; (3) history of hypertension or preeclampsia, thyroid disease, chronic kidney disease, autoimmune disease, or a diagnosis of these diseases in the current pregnancy; (4) pregestational diabetes mellitus; (5) long-term use of aspirin or glucocorticoids. This study was approved by the Ethical Committee of Guangzhou iBorn Women’s Hospital, and each patient signed the written informed consent.

A Voluson E8 ultrasound machine (GE Medical Systems, Milwaukee, WI, USA) with a 1–6 MHz transabdominal RAB6-1D probe was used in this study. The ultrasound scan was performed according to the ISUOG practice guidelines (16). The nuchal translucency (NT), biparietal diameter (BPD), CRL, abdominal circumference (AC), femur length (FL), uterine artery doppler pulsatility index (UTPI), uterine artery resistance index (UTRI), VI, FI, and VFI were measured between 11^+0^ and 13^+6^ gestational weeks.

**Figure 1:**
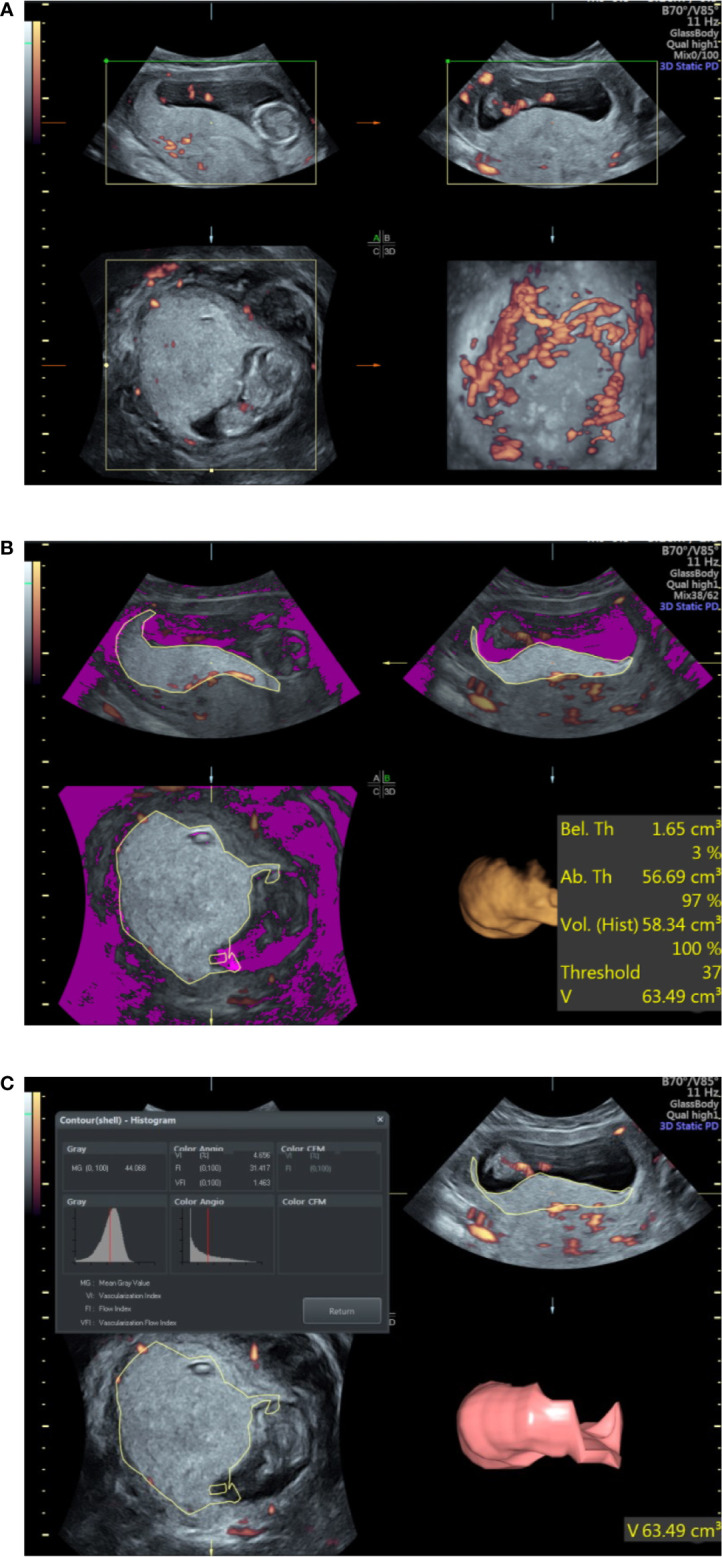
Ultrasound examination of the placental volume and vascular perfusion. **(A)** Placental volume as determined by three-dimensional power Doppler. **(B)** Measurement of placental volume by VOCAL. **(C)** Placental vascular indices determined by three-dimensional power Doppler.

To obtain optimal PV, the probe was placed along the alignment of the placenta. Each woman was asked to hold her breath for 10 s, and the margin of the placenta was outlined to obtain its maximum area. This procedure was repeated six times after rotating the probe 30 degrees around the axis each time to acquire the full volume of the placenta. For laterally and posteriorly located placentas, the position of the probe was adjusted to fit the placental alignment as far as possible to obtain the optimal volume (**Figure 1**). All of the examined cases were measured using the same ultrasound instrument settings (Power Doppler map, 6; frequency, low; smoothing, rise 2/fall 4; flow resolution, mild 1; line density, 7; balance, 210; ensemble, 10; line filter, 3; artifact suppression, on; quality, high1; wall motion filter, med 1; pulse repetition frequency, 1.3 kHz). All the ultrasound scans were performed by the same examiner, and the placental vascular indices and PV were measured twice to inspect the intrarater reliability.

The three vascular indices (VI, FI, and VFI) were developed through specific algorithms based on signal intensity and the relative proportion of color voxels (three-dimensional pixels) within the defined volume, where VFI is a combination of VI and FI information (17). The placental vascular indices were automatically calculated using VOCAL™ imaging software (GE Medical Systems) and expressed on a scale of 0–100 (**Figure 1**).

All pregnant women were followed up after delivery. Screening for GDM was universally performed at 24–28 gestational weeks according to Guideline for Diagnosis and Treatment of Gestational Diabetes Mellitus (2014) (6). The GDM is diagnosed if any of the oral glucose tolerance test plasma glucose values meets or exceeds the following cut-off values: 5.1 mmol/L at fasting; 10.0 mmol/L at 1 h; and 8.5 mmol/L at 2 h. The follow-up data were collected as follows: pregnant complications, gestational age, modes of delivery, 1 and 5 min Apgar scores, birth weight, placental weight, and placental volume after delivery. The placental weight and placental volume after delivery were determined according to previous methods (18).

All the data analyses were performed using SPSS software (version 22.0, IBM, Armonk, USA). The normality of the data was examined by the Kolmogorov-Smirnov test. The Chi-square test was used for categorical variables. The Student’s t-test was used to evaluate differences in the continuous data between non-GDM and GDM group, and the continuous data were presented as mean ± standard deviation. Univariate and multivariate logistic regression analyses of individual confounding factor were also performed. Receiver operating characteristic (ROC) and the area under the ROC curve (AUC) were applied to evaluate the diagnostic value and the Youden index is a measure of a diagnostic test’s ability to balance sensitivity (detecting disease) and specificity (detecting health or no disease) (19). Youden index was applied to determine the optimal cut-off points of different parameters in the diagnosis of GDM. P < 0.05 was considered statistical significance.

## Results

### Clinical Characteristics of Pregnant Women in the Non-GDM and GDM Group

In this study, a total of 161 pregnant women received ultrasonographic examination. Among these pregnancies, four women had miscarriage during the second trimester; nine women had pregnant complications (hypertension, preeclampsia, or thyroid disease); seven women lost to follow-up. Thus, 106 pregnant women without GDM and 35 pregnant women with GDM were included in this study. As shown in **Table 1**, the maternal age and gravida in the GDM group were significantly higher than that in the non-GDM group (**Table 1**). For the ultrasonographic examination, no significant difference was detected in NT, BPD, CRL, AC, FL, UTPI, and UTRI between non-GDM and GDM group (**Table 1**). There was no significant difference in gestational age at delivery, Caesarean section, and premature birth rates between non-GDM and GDM group (**Table 1**). For the newborns, no significant difference was identified in birth weight, placental weight, PV after delivery, 1 and 5 min Apgar scores, between non-GDM and GDM group (**Table 1**).

**Table 1:** Comparison of clinical characteristics between non-GDM group and GDM group.

	Non-GDM group (n = 106)	GDM group (n = 35)	*P* value
**Maternal**			
Maternal age (years old)	30.04 ± 4.24	32.40 ± 4.56	0.004
BMI (kg/m^2^)	21.36 ± 2.54	22.34 ± 2.98	0.062
Gravida	1.88 ± 1.03	2.34 ± 1.37	0.035
Parity			
Primiparity	61	17	0.4336
Multiparity	45	18	
**Ultrasonography**			
NT (mm)	1.45 ± 0.40	1.35 ± 0.38	0.972
BPD (cm)	2.04 ± 0.26	2.03 ± 0.27	0.706
CRL (cm)	6.29 ± 0.82	6.06 ± 0.79	0.160
AC (cm)	6.19 ± 0.82	5.95 ± 0.86	0.128
FL (cm)	0.75 ± 0.20	0.72 ± 0.22	0.335
UTPI	1.43 ± 0.51	1.40 ± 0.45	0.776
UTRI	0.67 ± 0.13	0.64 ± 0.16	0.305
**Outcomes**			
Gestational age at delivery (weeks)	39.31 ± 1.32	38.89 ± 1.83	0.141
Caesarean section (%)	36.79% (39/106)	40.00% (14/35)	0.841
Premature birth (%)	5.66% (6/106)	2.86% (1/35)	0.681
Birth weight (g)	3203 ± 394	3229 ± 496	0.182
Placental weight (g)	561.50 ± 83.74	548.56 ± 68.31	0.471
Placental volume after delivery (cm^3^)	462.83 ± 123.65	462.55 ± 104.36	0.991
1 min Apgar score	9.95 ± 0.25	10.00 ± 0.00	0.275
5 min Apgar score	10.00 ± 0.00	10.00 ± 0.00	–

AC, abdominal circumference; BMI, body mass index; BPD, biparietal diameter; CRL, crown-rump length; FL, Femur length; GDM, gestational diabetes mellitus; NT, nuchal translucency; UTPI, uterine artery doppler pulsatility index; UTRI, uterine artery resistance index.

### Comparison of Placental Volume and Vascular Perfusion Between Non-GDM Group and GDM Group

**Figure 2:**
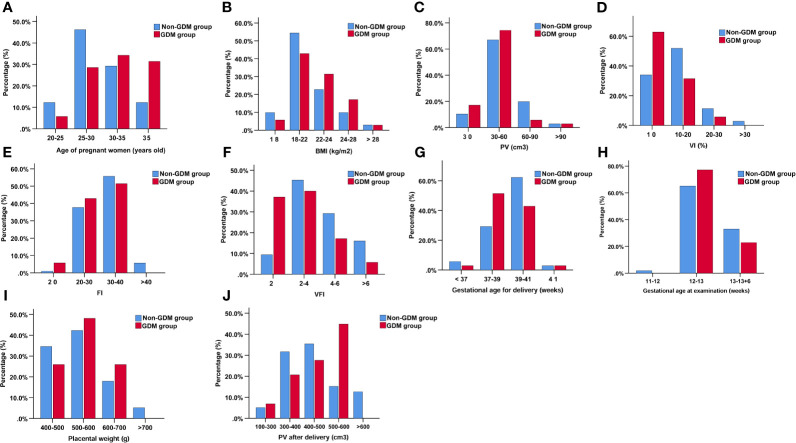
The distribution of pregnant women based on age of pregnant women **(A)**, BMI **(B)**, PV **(C)**, VI (%) **(D)**, FI **(E)**, VFI **(F)**, gestational age for delivery **(G)**, gestational age at examination **(H)**, placental weight **(I)**, and PV after delivery **(J)**.

The gestational age at ultrasound examination was 12.76 ± 0.48 and 12.64 ± 0.43 weeks in non-GDM and GDM group, respectively (**Table 2**). In the non-GDM group, PV (cm^3^), VI, FI, and VFI were 50.49 ± 18.53, 13.08 ± 6.30, 31.71 ± 5.71, and 4.15 ± 2.17, respectively (**Table 2**); in the GDM group, the PV (cm^3^), VI, FI, and VFI were 43.20 ± 14.07, 9.49 ± 6.46, 29.18 ± 5.46, and 2.82 ± 2.05 (**Table 2**). PV, VI, FI, and VFI in the GDM group were significantly lower than that in the non-GDM group (**Table 2**). The distribution of pregnant women based on maternal age, maternal BMI, gestational age at examination, PV, VI, FI, VFI, gestational age at delivery, placental weight, and PV after delivery was shown in **Figure 2**. In terms of maternal age, the distribution of maternal age was mainly between 25 and 35 years old; while the proportion of patients >35 years old was higher in the GDM group (**Figure 2**). The distribution of BMI, PV, and FI was mainly between 18 and 24 kg/m^2^, 30–60 cm^3^, and 20–40, respectively (**Figure 2**). In terms of VFI, the distribution of VFI < 2 in GDM group was higher than that in the non-GDM group (**Figure 2**).

**Table 2:** Comparison of placental volume and vascular perfusion between non-GDM group and GDM group.

	Non-GDM group (n = 106)	GDM group (n = 35)	*P* value
Gestational age at examination (weeks)	12.76 ± 0.48	12.64 ± 0.43	0.184
PV (cm^3^)	50.49 ± 18.53	43.20 ± 14.07	0.025
VI (%)	13.08 ± 6.30	9.49 ± 6.46	0.001
FI	31.71 ± 5.71	29.18 ± 5.46	0.023
VFI	4.15 ± 2.17	2.82 ± 2.05	< 0.001

FI, flow index; GDM, gestational diabetes mellitus; PV, placental volume; VFI, vascularization-flow index; VI, vascularization index.

### Univariate and Multivariate Logistic Regression Analyses of Clinical Parameters Associated With GDM

Univariate and multivariate analyses were performed to determine the association between clinical parameters and GDM. The univariate analysis showed that maternal age [odds ratio (OR) = 1.13, 95% confidence interval (CI) = 1.03–1.23, P = 0.008], gravida (OR = 2.55, 95% CI = 1.12–5.80, P = 0.0026), PV (OR = 0.97, 95% CI = 0.95–1.00, P = 0.038), VI (OR = 0.90, 95% CI = 0.83–0.97, P = 0.006), FI (OR = 0.92, 95% CI = 0.86–0.99, P = 0.026), and VFI (OR = 0.68, 95% CI = 0.53–0.87, P = 0.002) were significantly associated with GDM (**Table 3**). Multivariate analysis revealed that VI (OR = 0.89, 95% CI = 0.83–0.96, P = 0.003), FI (OR: 0.93, 95% CI = 0.86–1.00, P = 0.038), and VFI (OR = 0.67, 95% CI = 0.52–0.87, P = 0.002) were significantly associated with GDM (**Table 3**); while PV (OR = 0.98, 95% CI = 0.95–1.00, P = 0.076) had no effect on GDM (**Table 3**).

**Table 3:** Univariate and multivariate logistic regression analysis of clinical parameters associated with GDM.

Variables	Univariate analysis			Multivariate analysis		
	OR	95% CI	*P* value	OR	95% CI	*P* value
Age	1.13	1.03–1.23	0.008			
Gravida	2.55	1.12–5.80	0.026			
PV (cm³)	0.97	0.95–1.00	0.038	0.98	0.95–1.00	0.076
VI (%)	0.90	0.83–0.97	0.006	0.89	0.83–0.96	0.003
FI	0.92	0.86–0.99	0.026	0.93	0.86–1.00	0.038
VFI	0.68	0.53–0.87	0.002	0.67	0.52–0.87	0.002

CI, confidence interval; FI, flow index; OR, odds ratio; PV, placental volume; VFI, vascularization-flow index; VI, vascularization index.

### Diagnostic Values of PV, VI, FI, and VFI for GDM

**Figure 3:**
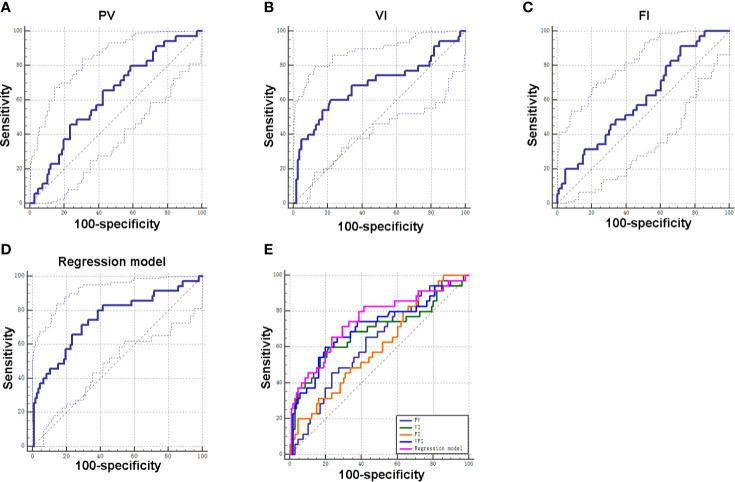
Diagnostic values of PV, VI, FI, and VFI for GDM. **(A)** ROC curve of PV for distinguishing GDM patients from non-GDM patients. **(B)** ROC curve of VI for distinguishing GDM patients from non-GDM patients. **(C)** ROC curve of FI for distinguishing GDM patients from non-GDM patients. **(D)** ROC curve of integrated factors using regression model for distinguishing GDM patients from non-GDM patients. **(E)** ROC curves of individual factor and integrated factors for distinguishing GDM patients from non-GDM patients.

Subsequently, ROC curves were used to evaluate the ability of PV, VI, FI, and VFI for diagnosing GDM. The ROC analysis revealed that PV, VI, FI, and VFI yielded an AUC of 0.63, 0.69, 0.61, and 0.71, respectively (**Table 4** and **Figure 3**). In addition, based on the Youden index, the results indicated that VFI (Youden index = 0.40) had the best diagnostic value for GDM when compared to VI (Youden index = 0.38), PV (Youden index = 0.23), and FI (Youden index = 0.20; **Table 4** and **Figure 3**). As maternal age and gravida were potential risk factors for GDM in our cohort, we combined maternal age, gravida, PV, and VFI by using the regression model. The ROC analysis revealed an AUC of 0.76 (95% CI = 0.66–0.86, P < 0.001; **Table 4** and **Figure 3**). ROC analysis indicated that the combination of maternal age, gravida, PV and VFI was more accurate as a marker for detecting GDM than the PV, VI, FI, or VFI alone (**Table 4** and **Figure 3E**).

**Table 4:** Diagnostic values of PV, VI, FI, and VFI for GDM.

Variables	AUC	Sensitivity (%)	Specificity (%)	Cut-off value	95% CI	LR+	LR–	Youden index	*P* value
PV (cm³)	0.63	65.71	57.55	44.90	0.52–0.73	1.55	0.60	0.23	0.017
VI (%)	0.69	60.00	78.30	8.22	0.57–0.80	2.77	0.51	0.38	0.002
FI	0.61	91.43	28.30	34.93	0.50–0.71	1.28	0.30	0.20	0.053
VFI	0.71	60.00	80.19	2.62	0.60–0.82	3.03	0.50	0.40	<0.001
Regression model	0.761	62.26	82.86	–	0.66–0.86	3.24	0.46	0.45	<0.001

AUC, area under the curve; FI, flow index; LR+, positive likelihood ratio; LR–, negative likelihood ratio; OR, odds ratio; PV, placental volume; VFI, vascularization-flow index; VI, vascularization index.

## Discussion

According to the demographic data, advanced maternal age is one of the risk factors for GDM. Schaefer et al. found that women older than 35 years old had 3.95-fold increased risk of GDM compared with women aged between 16 and 25 years old (20). A study by analyzing the prevalence of GDM in the USA between 2007 and 2014 indicated that older age was one of the risk factors associated with GDM (21). Shan et al., found that there was a 2–3-fold risk for mothers with advanced maternal age to be diagnosed with GDM in a retrospective cohort study from China (22). Khalil et al., analyzed a population of 22,933 pregnancies, and demonstrated that advanced maternal age was a risk factor for GDM (23). Our results consistently showed that the maternal age in the GDM group was higher than that in the non-GDM group, and maternal age was included in the subsequent ROC analysis.

In this study, we used the VOCAL to determine the PV and placental vascular indices (VI, FI, and VFI). Our results showed that the PV in the GDM group was significantly smaller than that in the non-GDM group during early pregnancy. However, Wong et al., showed that the PV was similar between GDM and non-GDM group during the first trimester, while it was significantly increased in the GDM group during the second trimester (14). Moreover, studies demonstrated that placental calcification and volume increased with advancing gestation in pre-gestational diabetic placentae (12, 24). On the other hand, no significant difference was detected in the PV and placental weight after delivery. These discrepancies may be to the small sample size and the variations in determine the PV after delivery, and future studies should include large samples to confirm our findings. In the analysis of VI, FI, and VFI, previous studies from Hafner et al., showed that VI, FI, and VFI could be used for a quick and reliable first trimester assessment of severe pregnancy risks (25). Consistently, Wong et al., showed that VI, FI, and VFI were significantly lower in the GDM group during the first and second trimesters (14). In addition, VI, FI, and VFI were significantly lower in diabetic pregnancies between 35 and 40 gestational weeks (24). Consistently, our results showed that the above three vascular indices were significantly lower in the GDM group during the early pregnancy. The above results indicated that there was a remarkable reduction in VI, FI, and VFI during GDM.

Based on the logistic regression analysis, our results indicated that reduced PV, VI, FI, and VFI were associated with the increased risk of GDM, which was consistent with the findings from Wong et al. (14). In addition, PV and vascular indices during early pregnancy could be used to predict the pregnant complications (25). Although the PV increases with the advancement in gestational age, the placental vascular index of pregnant women with GDM still decreases during the third trimester (14). This finding may be related to the abnormal secretion of key mediators in GDM pregnant women in the first trimester, which affects placental vascular remodeling, and finally affects the placental development, leading to increased PV and reduced vascular indices (26, 27). Moran et al., found that PV was significantly larger at all stages of gestation from 12 weeks (24). In our study, the PV was mainly determined between 11^+0^ and 13^+6^ weeks, when the changes in placenta have taken place, thus, suggesting the reliability of our findings.

This study also determined diagnostic potentials of PV, VI, FI, VFI in GDM. According to the results of the ROC curve of each parameter, the AUC of VFI curve is the largest, indicating good predictive value, and its sensitivity and specificity are 60.00 and 80.19%, respectively, which means that the diagnosis rate is high and the missed diagnosis rate is low. In addition, Youden index is the highest for VFI, indicating that it has good clinical practical value. The AUC values of PV, VI, and FI were less than 0.7, indicating that its predictive value is low. FI has the highest sensitivity, but low specificity, which indicates that its misdiagnosis rate is high. Our results were consistent with previous studies showing that VFI may be a more sensitive sonographic marker than VI and FI in the first trimester of GDM placentas (14). Our results of combined maternal age, gravida, PV, and VFI by using the regression model revealed that the combination of these parameters was more accurate as a marker for detecting GDM than the PV, VI, FI, or VFI alone, suggesting that the maternal age, gravida, PV, and VFI may representative important diagnostic markers for GDM.

This study is subjected to several limitations. First of all, the present study is limited to small sample size, and larger population of pregnant women may be included for analysis in the future. Secondly, this study was a single-center prospective study, which may lead to study bias. Multiple-center studies should be considered, in order to confirm our findings. Thirdly, how PV and vascular indices correlate with GDM remains unknown, which still requires the mechanistic investigations.

In conclusion, the 3D ultrasonography results suggest that PV and vascular indices (VI, FI, and VFI) during the first trimester may serve as potential markers for GDM diagnosis. The combination of maternal age, gravida, and sonographic markers may have good diagnostic values for GDM, which should be confirmed by further investigations.

## Data Availability Statement

The original contributions presented in the study are included in the article/supplementary material. Further inquiries can be directed to the corresponding authors.

## Ethics Statement

This study was approved by the Ethical Committee of Guangzhou iBorn Women’s Hospital, and each patient signed the written informed consent. The patients/participants provided their written informed consent to participate in this study.

## Author Contributions

HH, YY, and W-HC conceptualized the study and were involved in study design. ZH and YZ collected the data and performed the follow-up. XL and W-HC performed the data analysis. ZH and YZ drafted the manuscript and HH revised it critically. All authors contributed to the article and approved the submitted version.

## Conflict of Interest

The authors declare that the research was conducted in the absence of any commercial or financial relationships that could be construed as a potential conflict of interest.
